# Global isoform-specific transcript alterations and deregulated networks in clear cell renal cell carcinoma

**DOI:** 10.18632/oncotarget.25330

**Published:** 2018-05-04

**Authors:** Michael J. Hamilton, Thomas Girke, Ernest Martinez

**Affiliations:** ^1^ Department of Biochemistry, University of California at Riverside, Riverside, CA, USA; ^2^ Department of Botany and Plant Sciences, University of California at Riverside, Riverside, CA, USA

**Keywords:** kallisto, sleuth, transcript, ccRCC, cancer

## Abstract

Extensive genome-wide analyses of deregulated gene expression have now been performed for many types of cancer. However, most studies have focused on deregulation at the gene-level, which may overlook the alterations of specific transcripts for a given gene. Clear cell renal cell carcinoma (ccRCC) is one of the best-characterized and most pervasive renal cancers, and ccRCCs are well-documented to have aberrant RNA processing. In the present study, we examine the extent of aberrant isoform-specific RNA expression by reporting a comprehensive transcript-level analysis, using the new kallisto-sleuth-RATs pipeline, investigating coding and non-coding differential transcript expression in ccRCC. We analyzed 50 ccRCC tumors and their matched normal samples from The Cancer Genome Altas datasets. We identified 7,339 differentially expressed transcripts and 94 genes exhibiting differential transcript isoform usage in ccRCC. Additionally, transcript-level coexpression network analyses identified vasculature development and the tricarboxylic acid cycle as the most significantly deregulated networks correlating with ccRCC progression. These analyses uncovered several uncharacterized transcripts, including lncRNAs *FGD5-AS1* and *AL035661.1*, as potential regulators of the tricarboxylic acid cycle associated with ccRCC progression. As ccRCC still presents treatment challenges, our results provide a new resource of potential therapeutics targets and highlight the importance of exploring alternative methodologies in transcriptome-wide studies.

## INTRODUCTION

Renal cancer is one of the ten most frequently occurring cancers found in both males and females in the United States [1]. In 2018, an estimated 65,340 new cases of renal cancer will be diagnosed within the US with ~96% of them being renal cell carinomas (RCC) [2]. Most RCC tumors originate from the epithelial cells of proximal tubules within the cortex of the kidney, and RCCs carry with them several therapeutics challenges [3, 4]. Specifically, both chemotherapy and radiation treatments are largely ineffective, patients can be frequently asymptotic, and metastatic RCC has a relatively high 5-year mortality rate of > 90% [5]. Among the four major histological RCC subtypes, clear cell renal cell carcinoma (ccRCC) is the most common, observed within 75% of cases [6].

One of the characteristic features of ccRCC is the frequently mutated von Hippel-Lindau (VHL) gene, found within ~50% of ccRCC tumors, or loss of the short arm of chromosome 3 [7–10]. Loss of a functional VHL protein, a E3 ubiquitin ligase, results in enhanced stability of a family of transcription factors, known as hypoxia inducible transcription factors (HIFs) [11]. As a result of elevated HIF proteins, changes to expression levels of several HIF responsive genes can occur, such as vascular endothelial growth factor (*VEGF*), MET proto-oncogene (*c-MET*), and transforming growth factor (*TGF*), altering the pro-angiogenic, invasive and proliferative characteristics of cancer cells. With the advent of large-platform and high-throughout techniques, we have greatly improved our understanding of the VHL/HIF pathway, and we have expanded beyond this classical model to reveal other key molecular events that occur in ccRCC. In a recent comprehensive study examining ccRCC, an integrative pathway analysis showed one of the most frequently mutated subnetworks were genes that influence the epigenetic landscape, such as *PBRM1* and genes in the PBAF SWI/SNF chromatin remodeling complex [7].

However, despite the shift to global gene expression profiling, little attention has been given to examining transcript-specific changes in ccRCC and other cancers, possibly due to the additional computational constraints compared to conventional gene-level analyses. Aberrant transcript isoforms from altered transcription initiation, termination and RNA processing (including altered alternative splicing) are well-documented phenomena found within many cancers [8, 12–15]. Furthermore, abnormal RNA processing events can have profound effects on the function of coding and non-coding RNA species [16, 17]. In a recent example, inactivation of a histone methyltransferase, known as SET domain containing 2 (*SETD2*), was discovered to be one of the inciting causes of widespread transcriptional read-through and abnormal RNA chimera production found in ccRCC [16].

With the advent of alignment-free RNA-Seq quantification algorithms, larger scale and more comprehensive transcript-level analyses can now be performed with a smaller computational footprint. An example is kallisto, one of the fastest and most accurate transcript-level quantification programs. Instead of more time consuming read alignments, it uses a *k*-mer approach for quantifying the abundance of transcripts in RNA-seq experiments [18]. More recently, two R packages, sleuth and RATs (Relative Abundance of Transcripts), were developed that exploit the bootstrap estimates from kallisto to identify events of differential transcript expression and differential transcript usage, respectively [19, 20]. Differential transcript expression (DTE) is any change in the relative abundance of a transcript between two conditions. Alternatively, differential transcript usage (DTU) is the proportional change of the transcripts that a gene encodes. For example, DTU can frequently result in isoform-switching, in which the major isoform (most abundant) “switches” with an alternative transcript, and thereby that isoform is longer the major isoform of that particular gene. To our knowledge, there are relatively few transcriptome-level studies examining differential transcript expression in ccRCC, and these studies have either relied on microarray platforms or focused largely on one aspect of differential transcript expression (e.g. differential splicing) [21–26]. Importantly, transcript-level analyses can add greater resolution to a transcriptome-wide study, as significant DTE can evade traditional gene-level analysis techniques.

**Figure 1:**
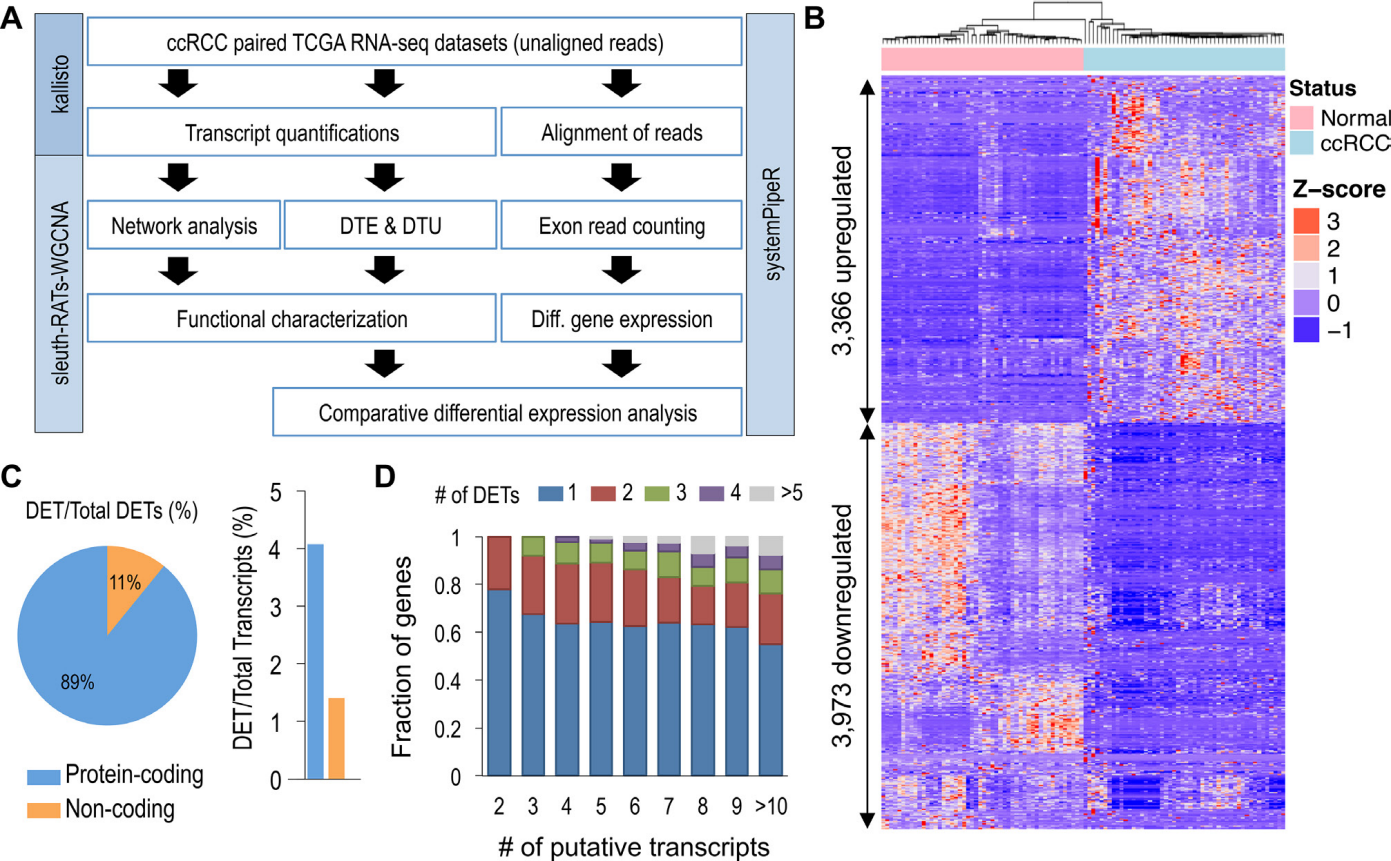
Global identification of differential transcript expression in ccRCC (**A**) Overview of pipeline used in identification and characterization of DTE and DTU in ccRCC. (**B**) Unsupervised hierarchical clustering of 7,339 DETs identified using sleuth (FDR < 0.005 and beta value of < -1 or > 1). (**C**) Percentage of protein-coding and non-coding genes for the 7,339 DETs identified using sleuth. (**D**) Proportion of genes with *n* identified DETs relative to total number of encoded transcripts.

The current study uses a multifaceted approach with new highly accurate computational methods, not employed by previous studies, quantifying all transcript-level alterations in ccRCC, and places these alterations in the context of key biological pathways involved in ccRCC progression (Figure 1A). In doing so, we identified several previously uncharacterized deregulated genes implicated in ccRCC. We analyzed 100 RNA-seq datasets (50 matched pair samples) from The Cancer Genome Altas (TCGA) with kallisto to quantify all putative coding and non-coding transcripts, sleuth to identify significant differentially expressed transcripts (DETs) and RATs to discover events of differential transcript usage (DTU). We identified 7,339 DETs and 94 DTU genes of which 68 genes are uncharacterized. Furthermore, we performed a comparative differential expression analysis, using both gene-level and transcript-level analyses, and identified novel deregulated genes in ccRCC. Additionally, we performed one of the first weighted transcript-level coexpression network analyses in ccRCC. Using WGCNA, we found that transcript networks controlling vascular development and TCA cycle were most significantly deregulated and correlated with ccRCC tumor stage. These analyses identified several uncharacterized genes as potential modulators of pathways deregulated in ccRCC.

## RESULTS

### Global identification and validation of DTE in ccRCC

From the kallisto analysis, a total of 217,082 transcripts quantifications (160,717 protein-coding and 56,365 non-coding) for each of the 100 samples were used in the differential expression analyses, comparing 50 normal adjacent renal samples against 50 ccRCC samples. Using the Wald test, with a log_2_ transformation, 90,002 transcripts passed the initial filtering process used by the sleuth R package. With a *q*-value of < 0.005, we identified 32,642 DETs, encoded by 14,767 genes (Supplementary Table 1, Supplementary Figure 1). With additional filtering, using the bias estimator, referred to as the beta value of > 1 or < -1 and an average absolute transcript expression of > 1 TPM, 7,339 high confidence DETs were identified (Figure 1B, Supplementary Table 1).

Gene ontology analyses using the express analysis in Metascape of the unique genes encoding the DETs are consistent with previous reports (Supplementary Table 1) [25, 27]. There is significant enrichment of gene sets and GO terms related to the immune response for the 3,366 upregulated DETs (encoded by 2,023 genes). Conversely, there is enrichment in GO terms related to metabolic processes and transport of small molecules and ions for the 3,973 downregulatd DETs (encoded by 2,518 genes). Previously reported and contained within the 7,339 DETs, is ras-related C3 botulinum toxin substrate 1 (*RAC1*), that shows a statistically significant downregulation of one of its transcripts, ENST00000356142.4 (Supplementary Figure 2) [13]. ENST00000356142.4 contains an additional exon, referred to as exon 3b that is frequently spliced out in ccRCC. The most abundant *RAC1* transcript, ENST00000348035.8, is unaffected in ccRCC.

As mutations in key epigenetic modifiers, such as *SETD2*, *PBRM1* and *BAP1*, among ccRCCs have demonstrated to have significant effects on the epigenetic landscape and consequently splicing events, we compared the DETs observed in the current study against 6,207 RefSeq transcripts previously found to have defects in splicing and intron retention [14]. Among the 6,207 transcripts, 6,070 transcripts were readily converted to an ensembl annotation, and 1,857 transcripts were identified as differentially expressed. In a similar study, among 30 genes found to have a deficiency in H3K36me3 and *SETD2*-mediated alternative splicing [15], we found 27 of these genes to have at least one DET in the current study (using an FDR < 0.005).

Among the 7,339 DETs discovered (4,470 individual loci), ~89% were protein-coding (6,546 transcripts) and ~11% were non-coding (793 transcripts) (Figure 1C, left). These DETs represented only ~4% and ~1% of the total putative protein-coding and non-coding transcripts, respectively (Figure 1C, right). Further characterization of the DETs showed that the number of transcripts affected remained relatively static, regardless of the number of putative transcripts derived from a given gene (Figure 1D). With genes encoding ≥ 2 transcripts, > 80% of the genes had ≤ 3 detectable DETs.

**Figure 2:**
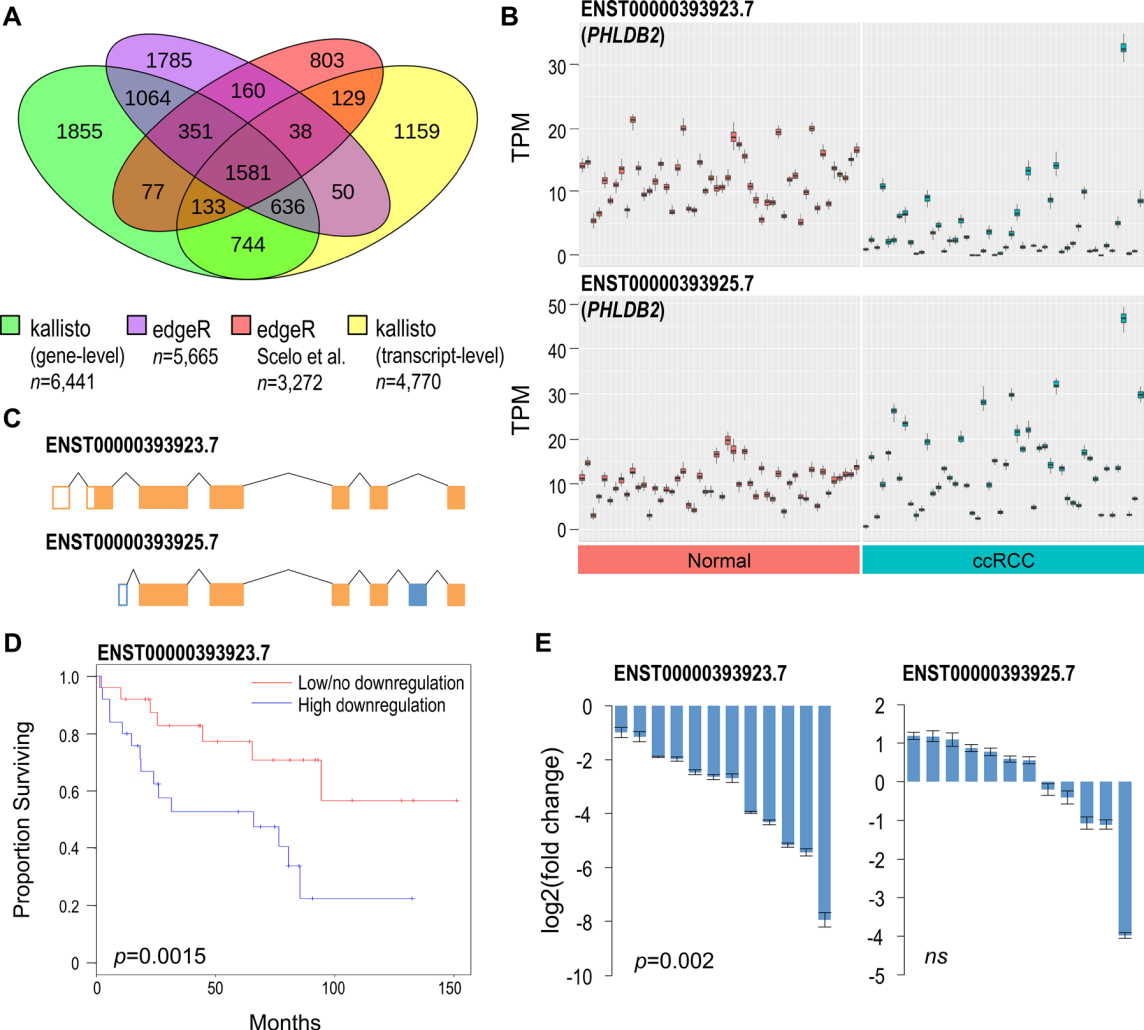
Comparative differential expression analysis identifies novel genes implicated in ccRCC (**A**) Comparison of DEGs/DTE genes discovered with sleuth, edgeR, and a previous study by Scelo et al. (**B**) Transcript abundances in normal renal and ccRCC tissues for the two most abundant *PHLDB2* transcripts. Each box plot represents 50 calculated bootstrap values of an individual sample (red = normal, blue = ccRCC). (**C**) ENST00000393923.7 harbors an alternative exon 1 and 2 and excludes exon 6 of ENST00000393925.7. Differences colored in blue. (**D**) Kaplan-Meier plot assessing survival of patients with high vs low/no ENST00000393923.7 downregulation. Median T/N ratio was used to partition samples into low/no and high downregulation groups. Log rank test was used to calculate statistical significance. (**E**) qPCR validation of *PHLDB2* DTE showing log2 fold change of 12 ccRCC tissues relative to their normal adjacent tissues. Results normalized to *PPIA* reference gene. Two-tailed Wilcoxon signed-rank test was used to determine statistical significance. Error bars = average standard deviation of technical replicates of pair samples. ns = non-significant (> 0.05).

Lastly, as previous gene-level expression analyses may not have detected some cases of DTE, we performed a comparative differential expression analysis of the matched pair samples evaluating the results of edgeR and sleuth [24] (Figure 2A). For the gene-level edgeR analysis, read counts were generated within the systemPipeR package, using HISAT2 for the alignment of the sequence reads and summarizeOverlaps for the generation of the gene counts. With thresholds of > 2 fold change and FDR < 0.005, edgeR identified 5,665 differentially expressed genes (DEGs). In an alternative gene-level analysis, using kallisto generated gene counts, the sleuth gene-level analysis discovered 6,441 DEGs, with a beta value of > 1 or < -1 and a FDR < 0.005. Among the 4470 genes, encoding the 7,339 DETs (described above), a total of 1,159 genes were found exclusively within the sleuth transcript-level analysis (Supplementary Table 1). Interestingly, only ~4% (51 genes) of the 1,159 genes harbored both upregulated and downregulated DETs. A moderate degree of overlap was observed between the four differential expression analyses, sharing 1,581 genes in common. Similarly, all gene-level analyses shared 1,932 genes in common, while the kallisto gene-level and our edgeR analyses had the most in common, sharing 3,632 DEGs.

One example of significant differentially expressed transcripts, not detected by gene-level analyses and not identified by previous ccRCC studies, are derived from Pleckstrin homology like domain family B member 2 (*PHLDB2*) known commonly for its association with vascular dementia (Figure 2B) [28]. *PHLDB2* encodes for 18 putative transcripts, and two transcripts ENST00000393923.7 and ENST00000431670.6 are downregulated in ccRCC (Supplementary Table 1). ENST00000393923.7 is the most abundant protein-coding *PHLDB2* transcript, and it is the most significantly downregulated in ccRCC (Figure 2C). ENST00000393925.7 is a slightly less abundant *PHLDB2* transcript, and it is unaffected in ccRCC. Evaluation of the tumor/normal TPM ratios of the 50 matched pair samples showed that patients with a high degree of ENST00000393923.7 downregulation exhibited lower survival rates over ~12 years (*p* = 0.0015, Figure 2D). Two additional examples of genes harboring DETs, solute carrier family 37 member 3 (*SLC37A3*) and high-density lipoprotein binding protein (*HDLBP*) were also found to correlate with patient survival (Supplementary Figure 3). ENST00000393923.7 downregulation was validated using transcript-specific qPCR with 12 independent matched pair ccRCC samples (Figure 2E). Using a Wilcoxon signed-rank test, ENST00000393923.7 was found to be significantly downregulated in ccRCC with a median downregulation of ~6.3 fold change. No statistically significant difference was observed with ENST00000393925.7.

### Weighted transcript-level coexpression network analysis

**Figure 3:**
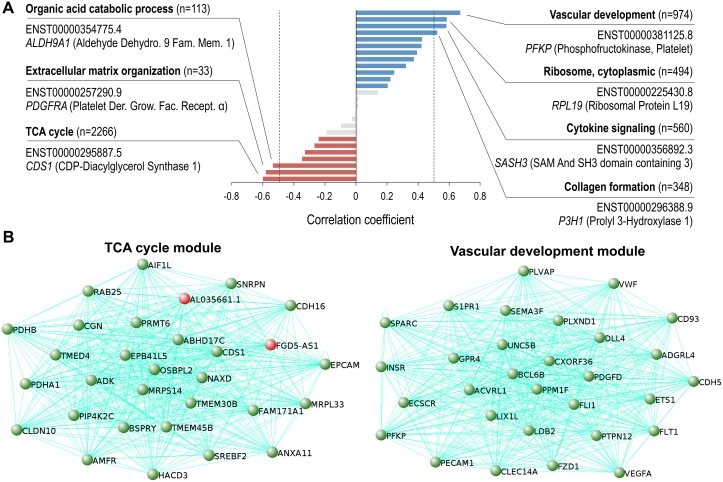
Vascular development and TCA cycle coexpression modules are the highest correlated networks in ccRCC progression (**A**) ccRCC correlated coexpression network modules identified with WGCNA. Using a correlation coefficient of > 0.5 or < -0.5 and *p* < 0.05, 4 positively correlated networks (blue bars, right of dotted line), and 3 negatively networks were identified to be in ccRCC (red bars, left of dotted line). Networks with no significant correlation with ccRCC (grey, *p* > 0.05). Most significant GO term for each module shown in bold, and the transcript with the highest module membership shown below. (**B**) Top 30 highest coexpressed transcripts (gene names shown) within the TCA cycle (left) and vascular development modules (right). Novel genes highlighted in red.

As our previous analyses suggest some transcripts derived from the same gene exhibiting different expression profiles, we sought to better understand the isoform-specific changes occurring within ccRCCs. Therefore, we pursued a weighted coexpression network analysis using the calculated transcript quantifications as a framework. Using WGCNA and the calculated TPM values from 10,000 of the most variable transcripts, a coexpression network was performed across five stages of ccRCC progression (normal, stage I, stage II, stage III, stage IV). A total of 26 coexpression modules were identified (Figure 3A), with 7 coexpression modules highly correlated with ccRCC progression (pearson coefficient > 0.5 or < -0.5 and *p* < 0.05). Using the Reactome, KEGG pathway, CORUM gene sets and the conventional GO terms, a Metascape analysis was performed separately with each of the 7 correlated coexpression modules. Among the 4 positively correlated coexpression modules, vascular development, ribosome, cytokine signaling and collagen formation were the most enriched terms found within each of the modules. Conversely, the 3 negatively correlated coexpression modules revealed TCA cycle, extracellular matrix organization and organic acid catabolic processes as the most significant terms (Supplementary Table 1). Identified within each of the modules were transcripts with the highest module membership, as these transcripts are likely extensively connected intramodular hubs (Figure 3A). These transcripts included: ENST00000381125.8 encoded by Phosphofructokinase, Platelet (*PFKP*), ENST00000356892.3 encoded by SAM And SH3 domain containing 3 (*SASH3*), ENST00000225430.8 encoded by Ribosomal Protein L19 (*RPL19*), ENST00000296388.9 encoded by Prolyl 3-Hydroxylase 1 (*P3H1*), ENST00000295887.5 encoded by CDP-Diacylglycerol Synthase 1 (*CDS1*), ENST00000257290.9 encoded by Platelet Derived Growth Factor Receptor Alpha (*PDGFRA*), and ENST00000354775.4 encoded by Aldehyde Dehydrodenase 9 Family Member 1 (*ALDH9A1*).

Further characterization of the coexpression networks showed that the majority of the transcripts comprising the networks, and all the transcripts used in the network construction, were encoded from separate individuals genes (Supplementary Figure 4). Additionally, validation of the network and gene set analyses showed 24 out of the top 30 coexpressed transcripts (transcripts with high adjacency scores) contained within the vascular development coexpression module, are derived from genes comprising the core signature angiogenesis genes described previously (Figure 3B, right) [29]. Moreover, among the top 30 coexpressed transcripts contained within the TCA coexpression module, 28 transcripts are produced by genes previously discovered as being downregulated in ccRCC (Figure 3B, left) [30]. The remaining transcripts, ENST00000424349.1 encoded by FGD5 antisense RNA 1 (*FGD5-AS1*) and ENST00000620459.1 encoded by *AL035661.1* are uncharacterized lncRNAs highly downregulated in ccRCC.

### Differential transcript usage in ccRCC

**Figure 4:**
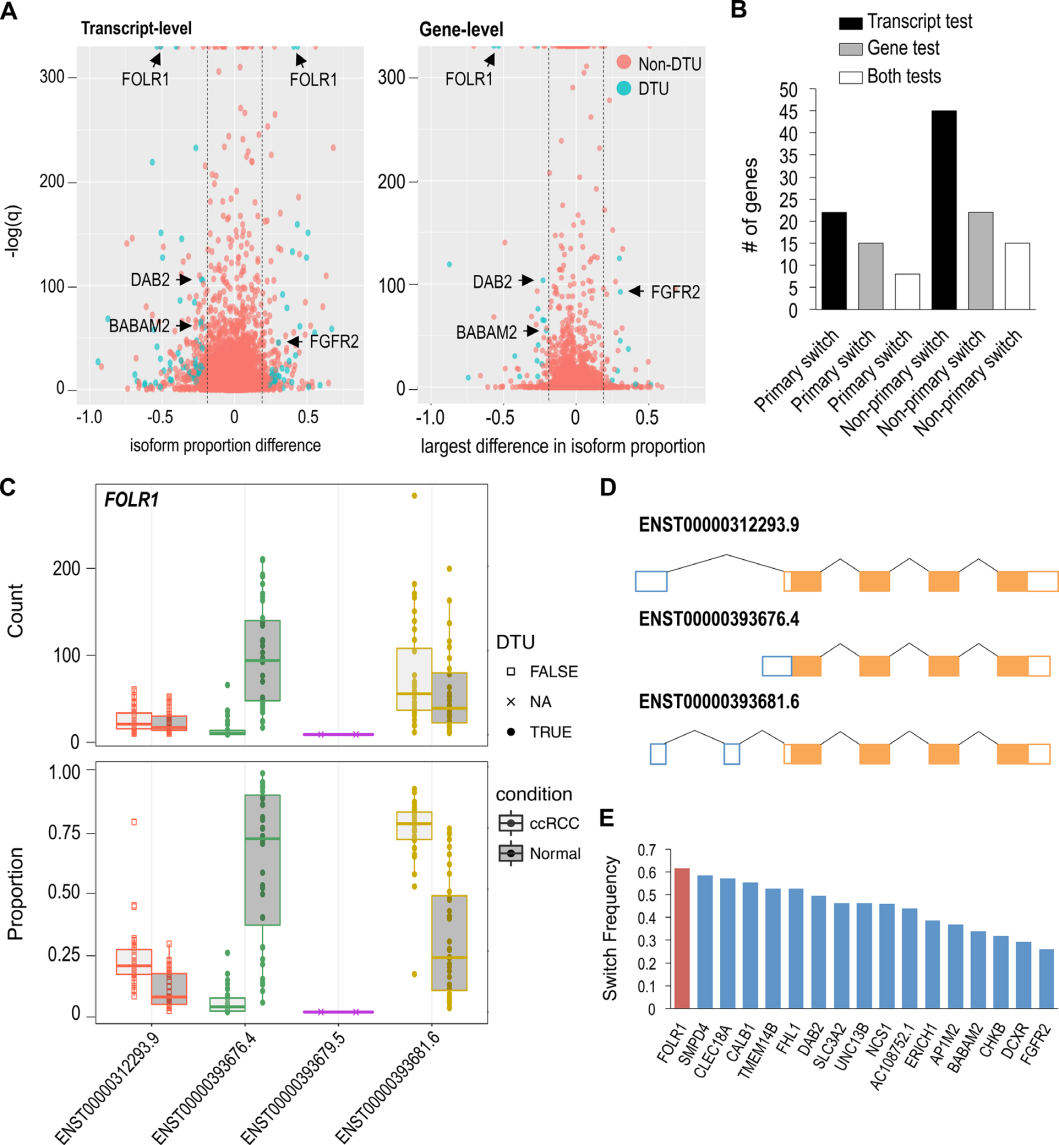
Few high frequency DTU genes observed in ccRCC (**A**) Transcript and gene-level tests using RATs to identify DTU events in ccRCC (red dot = non-DTU, blue dot = DTU). (**B**) Number of primary and non-primary isoform switches discovered in ccRCC. “Both” represents the number of shared DTU genes identified in both the transcript and gene-level tests. (**C**) *FOLR1* exhibiting significant proportional isoforms changes in ccRCC. Circle = significant DTU. Square = tested in DTU analysis, but not significant. X = did not meet abundance threshold for DTU anlaysis. (**D**) Schematic of *FOLR1* transcripts analyzed in DTU analysis. (**E**) Frequency of *FOLR1* and 17 other isoform switches shared between both DTU tests.

Using the kallisto transcript abundances, the RATs R package identified 97 events of differential transcript usage (Figure 4A, left, Supplementary Figure 5). These 97 transcripts were identified using the RATs transcript-level test, which examines each transcript individually and then merges the transcript information to form a gene-level finding. Alternatively, the gene-level DTU test, which collectively evaluates the transcripts of a gene, identified only 26 DTU genes (Figure 4A, right, Supplementary Figure 5). Among both transcript-level and gene-level DTU tests, 7 DTU genes (*AP1M2*, *CAB39L*, *CCDC146*, *C16orf89*, *DAB2*, *MAPK8IP1*, *FGFR2*) have been identified previously [25, 26]. Collectively, 94 DTU genes (68 uncharacterized DTU genes) in total were discovered (using both DTU tests) when comparing normal adjacent and ccRCC tissues (Supplementary Table 1). No statistically significant GO terms were enriched within the 94 DTU genes, using a corrected *p*-value. However, the Metascape analysis showed the top GO term (*p* = 0.0007) was carboxylic acid transport, supporting previous results demonstrating metabolic derangements as a cornerstone of ccRCC [7, 31]. Seven DTU genes were found to have a carboxylic acid transport GO classification, which included: *AGXT*, *SLC38A5*, *SLC9A4*, *SLC3A2*, *UNC13B*, *FABP6* and *FOLR1*.

Examination of the DTU events showed that non-primary (i.e. non-major) isoform switches are more frequent than primary isoform switches in ccRCC (Figure 4B). On average, we identified approximately twice as many non-primary isoform switches relative to primary isoform switches. Among the 8 primary isoform switches (in common between the DTU tests), all of them also had non-primary isoform switches. The DTU genes (described previously) *AP1M2*, *DAB2* and *FGFR2* exhibited both primary and non-primary isoform switching events (Supplementary Figures 6–7). Constituting the majority of DTU genes, a total of 76 DTU protein-coding genes were observed. The remaining DTU genes encompassed 11 ncRNA and 7 unclassified genes. Two examples of mostly uncharacterized DTU genes, with high isoform-switch frequencies, were *FOLR1* and *BABAM2* (Figure 4C, Supplementary Figure 6). *FOLR1,* known as folate receptor 1, produces 4 putative transcripts, and was found to be one of the most significant primary isoform switches. ENST00000393676.4 has an alternative 5’ end and is the most abundant *FOLR1* transcript in normal renal tissue (Figure 4D); however, ENST00000393681.6 switches with ENST00000393676.4 becoming the most abundant or primary *FOLR1* transcript in ccRCC. *FOLR1* had the highest isoform-switch frequency with 61% of ccRCC samples exhibiting the primary isoform-switch (Figure 4E). *BABAM2* encodes for a component of the BRCA1-A complex, and it produces 11 putative transcripts, 4 of which were eligible for DTU analysis. ENST00000436924.5 was the only *BABAM2* transcript to show a significant proportional increase in its abundance in ccRCC, becoming the second most abundant *BABAM2* transcript in ccRCC (Supplementary Figure 6).

## DISCUSSION

In the current study, we identified the global isoform-specific alterations in ccRCC and explored the deregulated networks implicated in ccRCC progression. Using the kallisto-sleuth pipeline, we discovered 7,339 DETs of which ~90% of the transcripts were derived from protein-coding genes. Additionally, comparative differential expression and coexpression network analyses aided in the discovery of several potentially clinically relevant genes and the major deregulated networks in ccRCC progression. Lastly, we discovered 68 uncharacterized high-frequency DTU genes in ccRCC with a suggested enrichment of genes involved in metabolic function.

Differential exon usage (DEU) has frequently been used as an inference for DTE in ccRCC [21, 23, 25]; however, this approach could present challenges in identifying DETs among transcripts sharing exons. Additionally, gene-level expression analyses could potentially overlook deregulated transcripts from clinically relevant genes that give rise to multiple transcripts. Therefore, we sought to identify deregulated transcripts and cognate genes that were not discovered readily by gene-level analyses by using novel methods that are not subject to the disadvantages of the DEU approach. In a typical gene-level analysis, all exonic reads from a gene are consolidated and used to determine if the expression of a gene is altered between two conditions. However, this approach could be disadvantageous in specific circumstances. One potential pitfall to a gene-level analysis is that if the other transcripts from the same gene are of similar abundance to the DET, then a conventional gene-level analysis may not detect a gene-level difference between the two conditions. Additionally, while isoform switching was found to be a relatively rare occurrence in ccRCC, isoform switching could also account for a “masking” of a relevant gene. *PHLD2, HDLBP* and *SLC37A3* are examples of this “masking” effect, in which DTE was not detected using conventional gene-level analyses. While we acknowledge that the degree of overlap between gene-level and transcript-level analyses could vary greatly depending on methodology and experimental thresholds, the current study highlights the importance of considering transcript-level analyses in comprehensive transcriptome-wide studies. Lastly, comparisons with previous studies, focused on *SETD2* mutational status/H3K36me3 prevalence of ccRCC tumors and the resulting effects on splicing [14, 15], suggest that genes subject to splicing defects can also harbor DETs. However, additional studies with large cohorts of mutation-specific ccRCCs are needed to determine isoform-specific expression changes that may be dependent on mutational status. As only 12 ccRCC tumors had a mutated *SETD2*, in the current study, our findings largely reflect *SETD2*-independent isoform-specific changes.

The discovery of two uncharacterized transcripts encoded by lncRNAs genes *FGD5-AS1* and *AL035661.1* identified in the network analysis suggest these lncRNAs transcripts could be potential regulators of TCA cycle genes or alternatively regulated by a common factor. These lncRNAs could be of particular importance to understanding ccRCC because of their implications in metabolic function. However, further investigation is needed, as the function of these lncRNAs is unknown. Another interesting transcript found within the TCA cycle coexpression module, identified with the highest module membership, is ENST00000295887.5 encoded by *CDS1. CDS1* encodes an integral membrane enzyme, located on the membranes of the mitochondrion and endoplasmic reticulum, that catalyzes the conversion of phosphatidic acid into CDP-diacylaglycerol [32, 33]. *CDS1* is uncharacterized in ccRCC and there is limited information on its role in cancer; however, in a recent study, *CDS1* was suggested to potentiate limitless growth and genomic instability in breast cancer [34].

We identified a total of 94 genes exhibiting differential transcript usage in ccRCC of which 7 DTU genes were reported previously [25, 26]. However, when considering the findings of an alternative study [24], which also evaluated lower frequency isoform-switches, the current study identified 26 DTU genes in common. Therefore, the differences observed in the DTU genes are likely attributed to different computational techniques/thresholds and/or the use of different transcript annotations [19]. While our findings show that the majority of isoform switching events involves non-primary isoforms, which is consistent with a previous result [24], alterations in the expression of non-primary isoforms could still be clinically relevant, as supported by the survival analyses seen with the non-primary *SLC37A3* and *HDLBP* deregulated transcripts. However, the mechanisms involved require further investigation. Recent studies have illustrated how isoform-specific alterations could be highly influential in ccRCC and other cancers. For instance, alternatively spliced isoforms of VHL were shown to alter VHL binding affinity to components of the p53 pathway [35]. Additionally, isoform-switching events have been demonstrated to alter the invasive properties of cancer cells [17, 36]. From our analyses and previous similar studies, mentioned above, it is highly suggestive that isoform-specific deregulations are a critical part to characterizing and understanding the molecular underpinnings of ccRCC, and suggest that isoform-level transcriptomic analyses should more generally be considered to obtain a more comprehensive view of the genetic deregulations in cancer.

## MATERIALS AND METHODS

### Transcript quantification and differential expression analyses

A total of 100 fastq RNA-seq files (50 primary ccRCC and 50 normal adjacent renal samples, Supplementary Table 1) were downloaded from The Cancer Genome Atlas (TCGA) legacy archive (https://portal.gdc.cancer.gov/legacy-archive/search/f). Human cDNA and ncRNA FASTA formatted transcript files (Ensembl v89 annotation) were acquired form the Ensembl ftp site (https://www.ensembl.org/info/data/ftp/index.html), and merged to create a master file of all putative coding and non-coding transcripts. All quantification and differential expression analyses were performed using the kallisto-sleuth pipeline. Using the default settings, kallisto was used to create an index for quantification using the aforementioned FASTA master file. Subsequently, kallisto was used to quantify all putative transcripts using 50 bootstrap samples. Differential expression analysis was performed with sleuth using the Wald test with a cutoff *q*-value of 0.005. RATs was performed using the read counts and bootstrap values calculated from kallisto. As ccRCC is a highly heterogeneous cancer, and there are 4 major subtypes of ccRCC, a replicate reproducibility of 0.25 was used in the analysis. All other parameters remained on default settings.

For the edgeR analysis, alignment of the fastq files was performed first with HISAT2 using the hg38 human assembly [37–39]. Read counting was performed using the summarizeOverlaps package, with union mode [40]. Using the read counts, an edgeR analysis was performed using the default settings. The entire pipeline was performed within the systemPipeR package [41].

### Weighted coexpression network analysis

All 217,082 TPM transcripts quantifications were initially filtered for an average absolute expression of > 1 TPM. Subsequently, 10,000 of the most variable transcripts, using the mean absolute deviation, were used for the proceeding WGCNA pipeline [42]. A soft thresholding power of 6 was used in a signed transcript coexpression network framework. All other parameters remained on the default recommended settings. ccRCC correlated coexpression networks were exported to VisANT with an adjacency threshold 0.08 for visualization purposes [43]. For the gene-level Metascape analysis (http://metascape.org) of each of the network modules, genes were considered only once in the analysis, regardless of the numbers of transcripts derived from the gene.

### Primer design and quantitiative PCR

Primers sequences were designed using Primer3 plus (http://primer3plus.com/cgi-bin/dev/primer3plus.cgi) using the default qPCR settings (Supplementary Table 1). When possible, primers were designed over exon junctions to avoid capturing unannotated alternative transcripts. All primers were synthesized by Integrated DNA Technologies. Twelve matched pair ccRCC RNA samples were acquired from Origene (Supplementary Table 1). Origene RNA samples were verified for quality and quantity using gel electrophoresis and the Thermoscientific Nanodrop2000 spectrophotometer. cDNA was synthesized using 1 ug of total RNA using the iScript reverse transcription supermix (Biorad, Irvine, CA) according to the manufacturer's instructions. Quantitative PCR was performed using the Biorad iQ SYBR green supermix and a Biorad CFX Connect thermocylcer (Biorad, Irvine, CA) and analyzed using the CFX manager software. Using a single threshold Cq determination, the Livak method was employed for all gene expression analyses. All qPCR analyses were normalized to *PPIA*, as *PPIA* was shown to be a suitable reference gene when comparing normal adjacent tissue to ccRCC tumor tissue [44, 45].

## SUPPLEMENTARY MATERIALS FIGURES AND TABLES





## Abbreviations

ccRCC: Clear cell renal cell carcinoma
TCGA: The cancer genome altas
WGCNA: Weighted correlation network analysis
RATs: Relative abundance of transcripts
DTE: Differential transcript expression
DTU: Differential transcript usage
DET: Differentially expressed transcript
