# Supplementary material for: Global isoform-specific transcript alterations and deregulated networks in clear cell renal cell carcinoma
Source: Oncotarget. 2018 May 4;9(34):23670–80. doi: 10.18632/oncotarget.25330 (PMC5955119; doi:10.18632/oncotarget.25330)
Supplement: Supplementary file 1 [file oncotarget-09-23670-s001.pdf]

Global isoform-specific transcript alterations and deregulated networks in clear cell renal cell carcinoma

SUPPLEMENTARY MATERIALS

Supplementary Table 1: Computational findings for all analyses and Supplementary Materials and Methods. See_Supplementary_Table 1

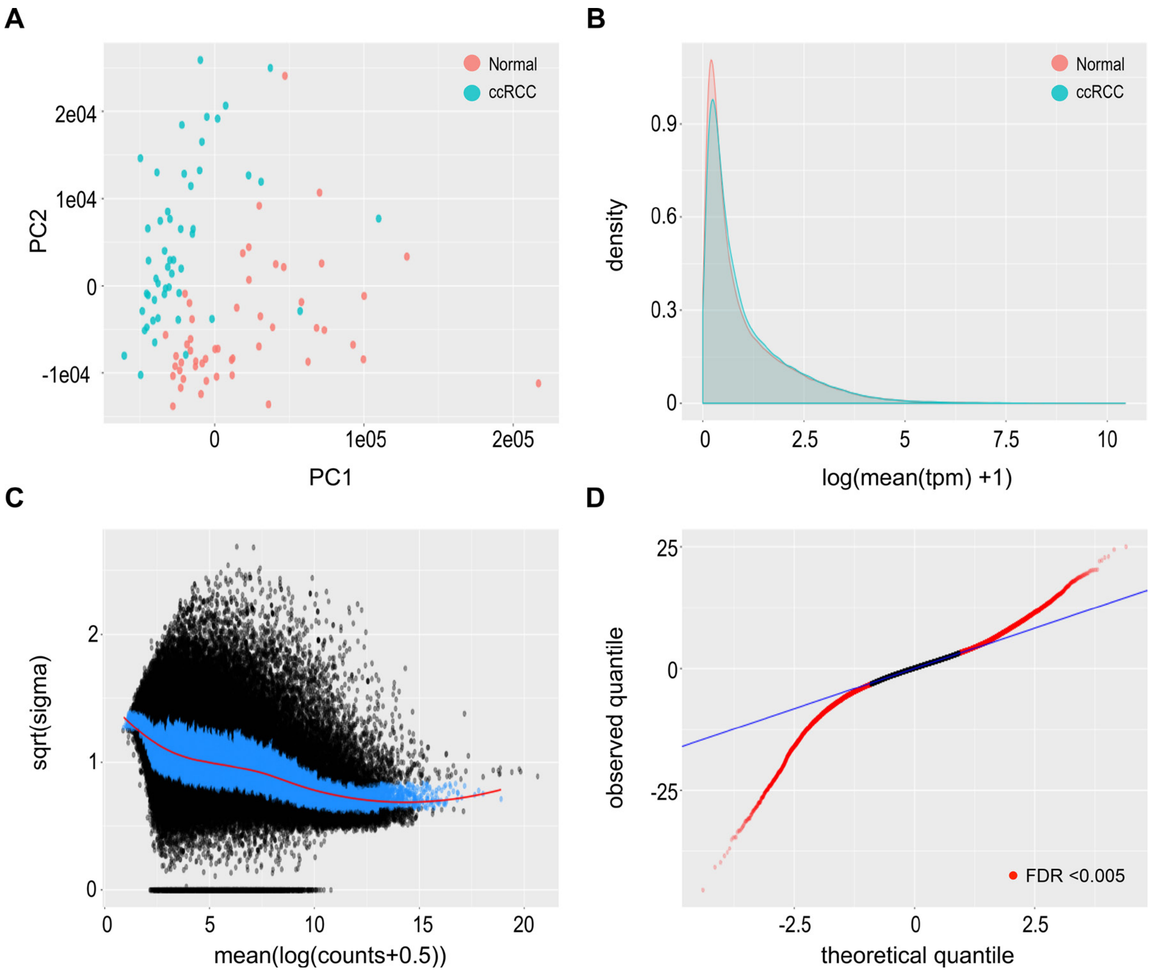

Supplementary Figure 1: Assessment of calculated transcript abundances and sleuth differential expression analysis. (**A**) Principal component analysis of TPM abundances to assess for outliers. (**B**) Distributions of transcript abundances by tissue status (normal vs ccRCC). (**C**) Mean-variance of transcripts modeled by sleuth (blue dots represent transcripts used in shrinkage estimation). (**D**) Q-Q plot assessing abundance distributions between normal and ccRCC samples (red dot = FDR < 0.005).

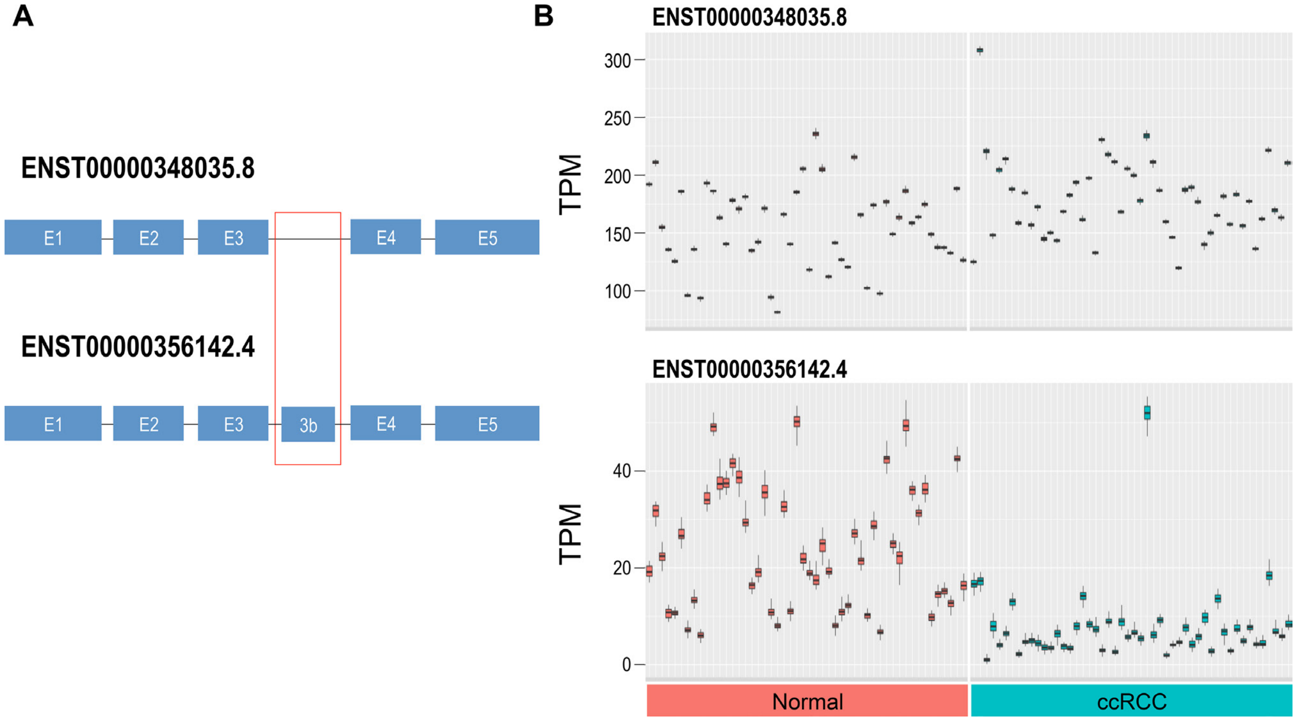

Supplementary Figure 2: ENST00000356142.4 (*RAC1*) transcript downregulated in ccRCC. (**A**) Schematic of most abundant protein-coding *RAC1* transcripts in normal renal tissue. ENST00000356142.4 contains an additional exon, referred as exon 3b (enclosed in red box). (**B**) *RAC1* transcript abundances in normal renal and ccRCC tissues. ENST00000356142.4 is downregulated in ccRCC. Each box plot represents 50 calculated bootstra*p* values of an individual sample (red = normal, blue = ccRCC).

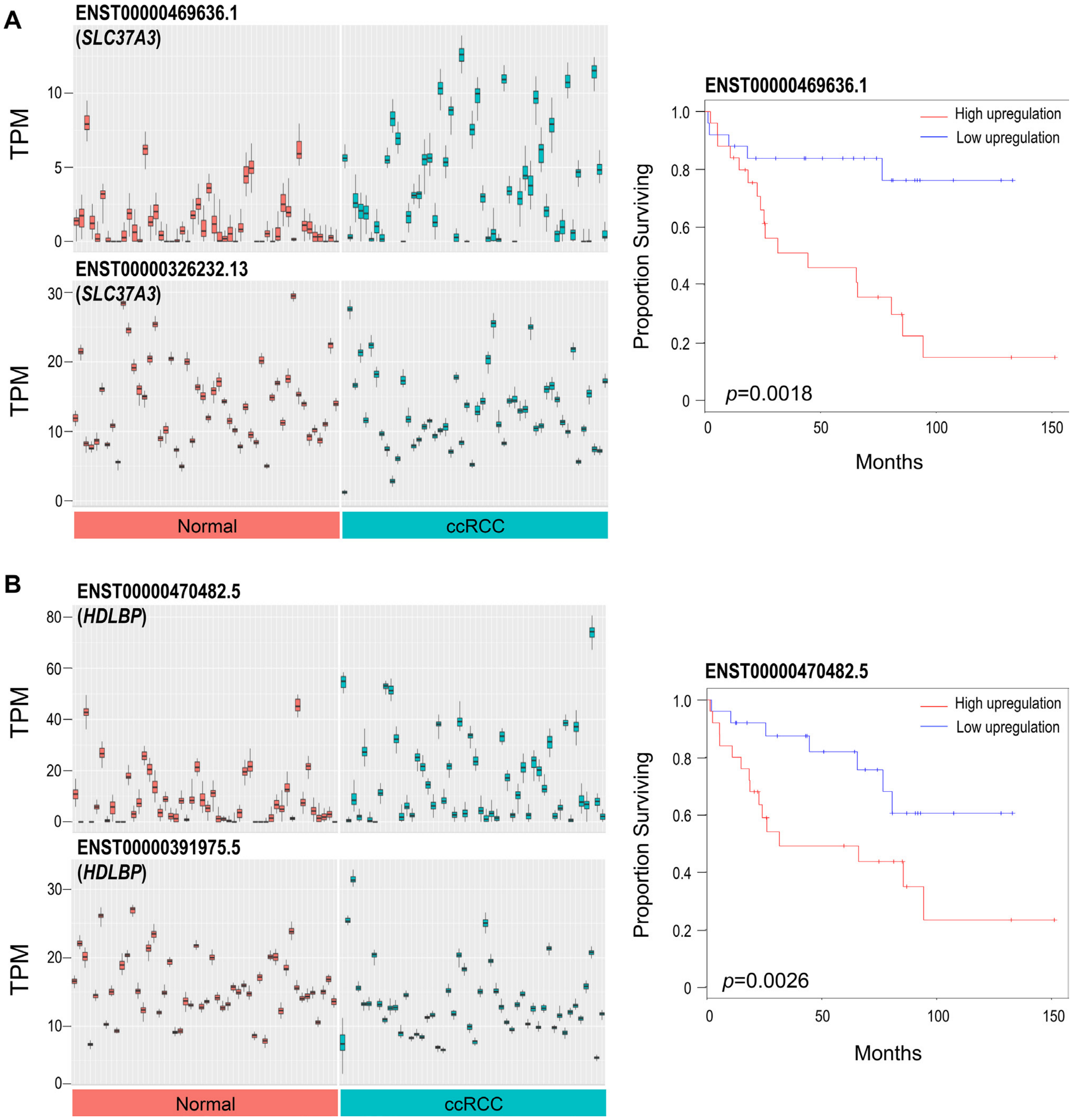

Supplementary Figure 3: *SLC37A3* and *HDLBP* upregulated transcripts in ccRCC. (**A**) *SLC37A3* transcript abundances in normal renal and ccRCC tissues (left). ENST00000469636.1 upregulated in ccRCC and correlated with patient survival (right). (**B**) *HDLBP* transcript abundances in normal renal and ccRCC tissues (left). ENST00000470482.5 upregulated in ccRCC and correlated with patient survival (right). Each box plot represents 50 calculated bootstrap values of an individual sample (red = normal, blue = ccRCC). Median T/N ratio was used to partition samples into low and high upregulation groups. Log rank test was used to calculate statistical significance.

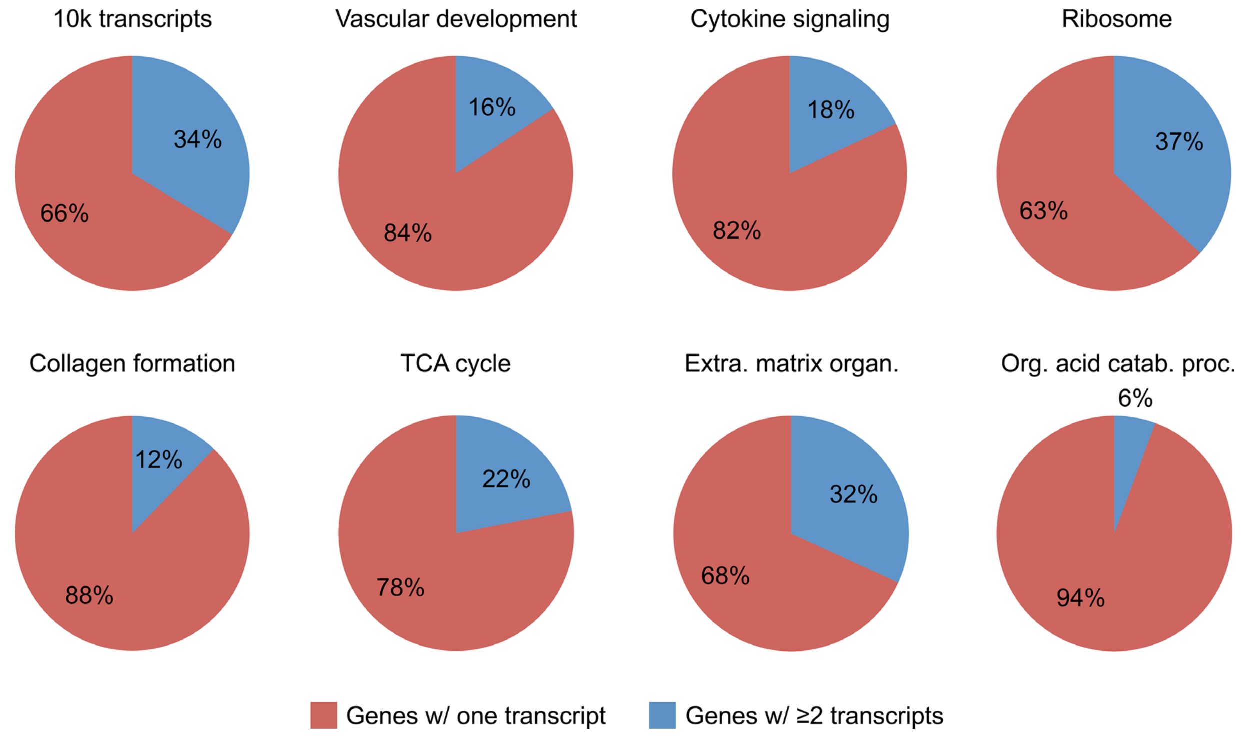

Supplementary Figure 4: Coexpression modules comprised mostly of transcripts encoded by unique genes. Assessment of transcripts used in the construction of the network analysis (top left) and the transcripts comprising ccRCC correlated modules. Red = genes with one transcript. Blue = gene with ≥ 2 transcripts.

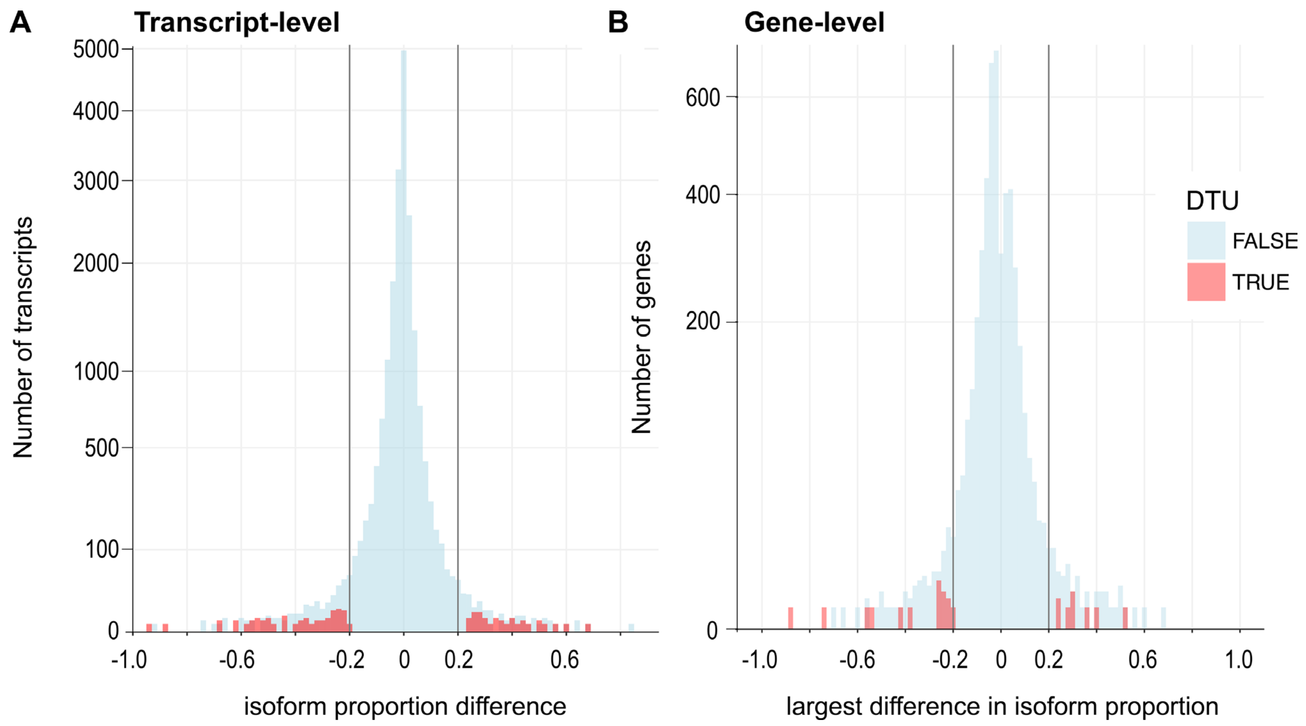

Supplementary Figure 5: Density distributions of DTU genes relative to isoform proportion differences. (**A**) Number of DTU transcripts and their isoform proportion differences discovered using RATs. (**B**) Number of DTU genes and their isoform proportion differences discovered using RATs. Red bar = DTU transcript/gene. Blue bar = non-DTU transcript/gene. Y-axis is square root compressed..

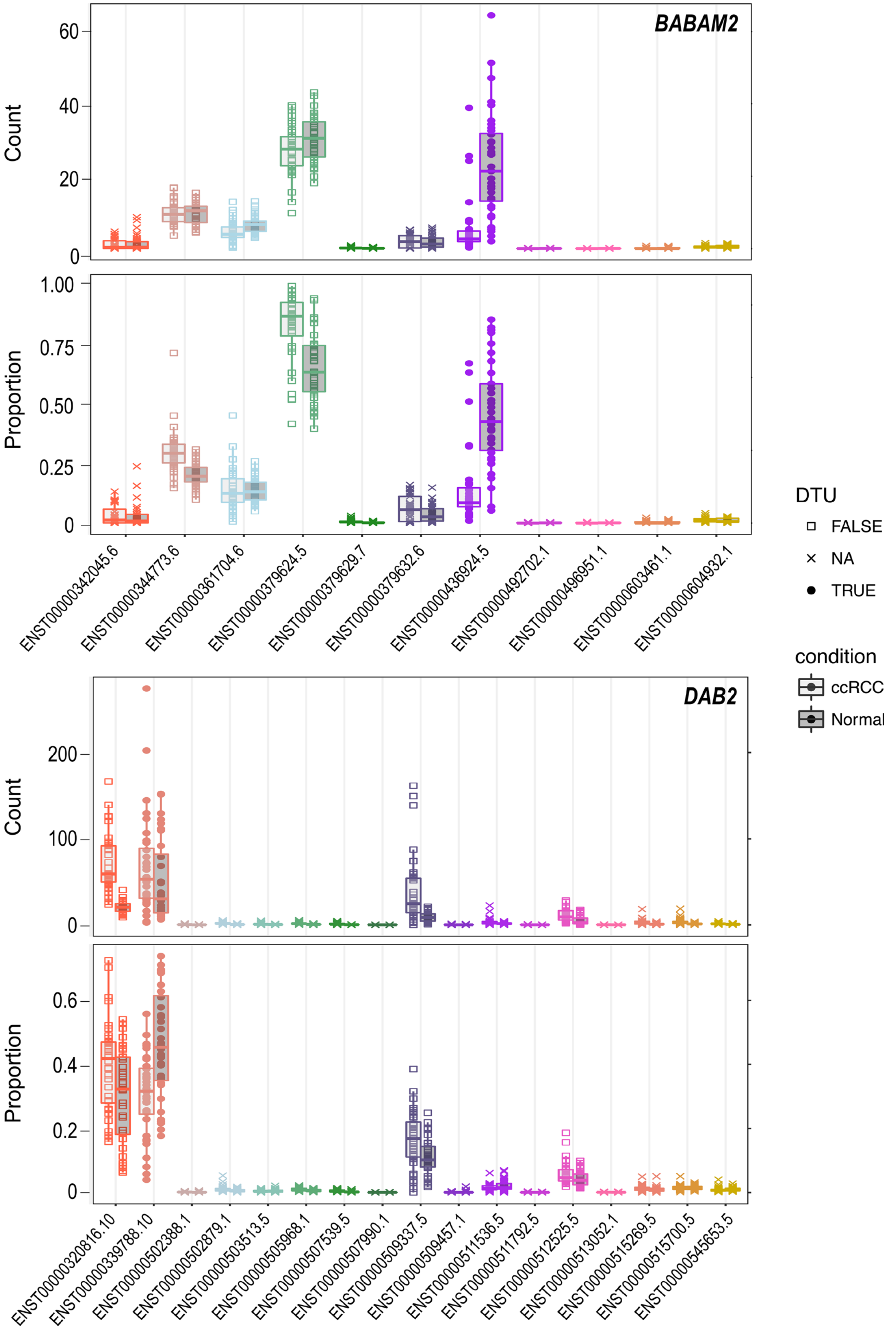

Supplementary Figure 6: *BABAM2* and *DAB2* DTU in ccRCC. Significant proportional increase observed in BABAM2 isoform ENST00000436924.5 in ccRCC (top). Significant proportional increase observed in DAB2 isoform ENST00000339788.10 in ccRCC. Circle = significant DTU. Square = tested in DTU analysis, but not significant. X = did not meet abundance threshold for DTU analysis.

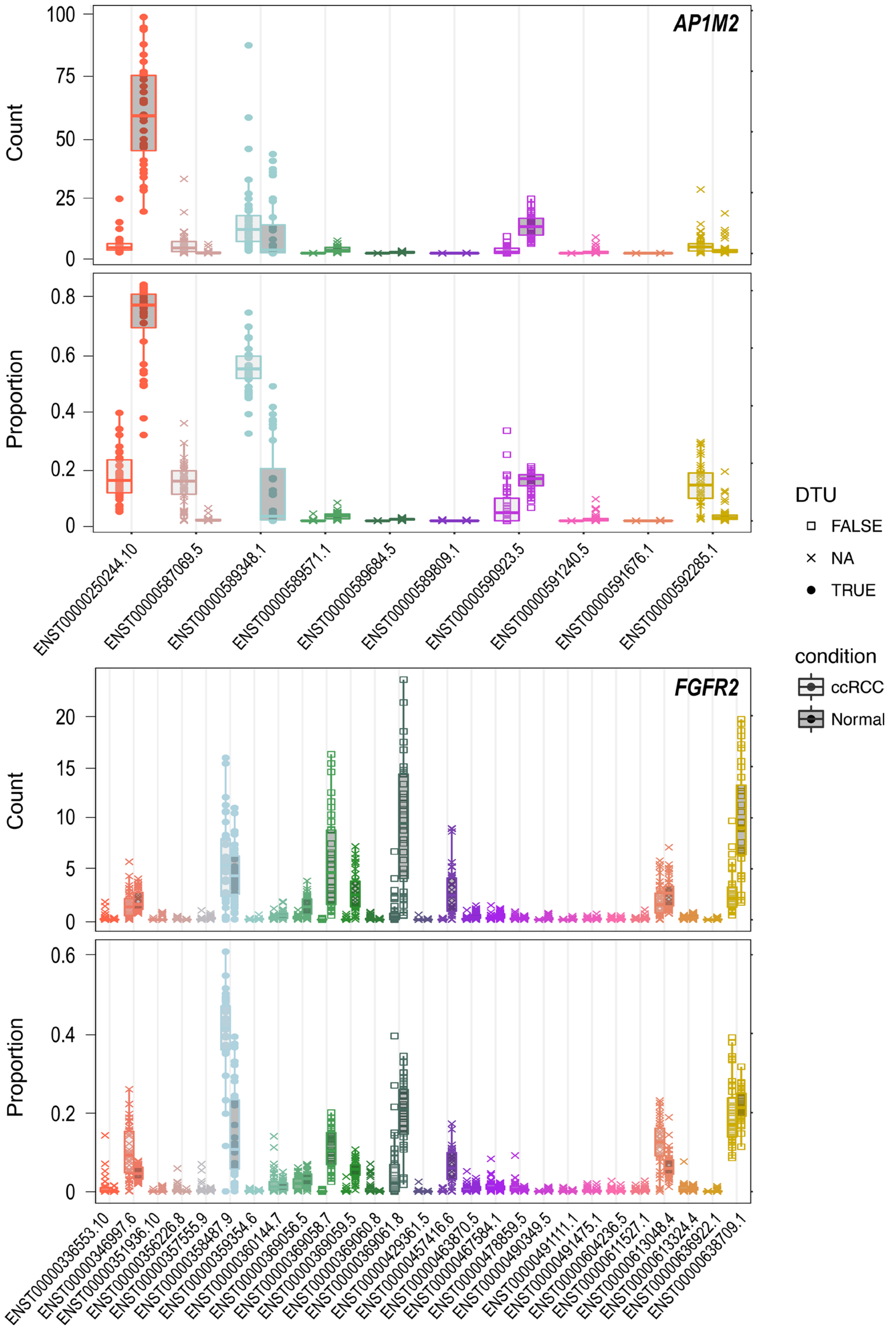

Supplementary Figure 7: *AP1M2* and *FGFR2* DTU in ccRCC. Primary isoform switch observed in *AP1M2* in ccRCC (top). A significant proportional decrease observed in *FGFR2* isoform ENST00000358487.9 in ccRCC. Circle = significant DTU. Square = tested in DTU analysis, but not significant. X = did not meet abundance threshold for DTU analysis.
